# Rural sixth-grade teachers’ and students’ perceptions of a mindfulness-based mental health curriculum

**DOI:** 10.3389/fpsyg.2023.1277614

**Published:** 2023-12-01

**Authors:** Austin Folger, Akhila Nekkanti, Gina Williamson, Claire Guidinger, Nichole R. Kelly

**Affiliations:** ^1^Counseling Psychology and Human Services, University of Oregon, Eugene, OR, United States; ^2^Choice Filled Lives Network, Inc., Berkeley, CA, United States; ^3^Prevention Science Institute, University of Oregon, Eugene, OR, United States

**Keywords:** acceptability, feasibility, mindfulness, rural, students

## Abstract

**Introduction:**

Mindfulness-based interventions (MBIs) have the potential to improve students’ mood, behavior and cognitive functioning; yet, little is known about the feasibility and acceptability of adapting such programs for rural middle schools.

**Methods:**

An exploratory qualitative evaluation was conducted to examine the feasibility and acceptability of an initial trial delivery of *AttuneEd*^®^, a trauma-informed, mindfulness-based mental health curriculum. In this single-group design study, 10 weekly lessons were taught in a middle school located in a rural town in the pacific northwest during 6th grade students’ P.E. classes. Three P.E. teachers, 26 6th grade teachers, and one school counselor attended trainings before and mid-curriculum implementation, where they provided qualitative feedback. A total of 160 students completed acceptability surveys before and after curriculum delivery.

**Results:**

Three themes were identified from qualitative data: cultural considerations, teacher self-efficacy, and barriers and facilitators to student acceptability. Student acceptability ratings were high. Students reported, on average, that the classes helped them better understand themselves and others.

**Conclusion:**

Some identified needs for future MBIs include (1) the need for culturally sensitive, trauma-informed delivery strategies; (2) teachers’ desire for more support in content delivery; and (3) students’ desire to have their own teachers deliver the curriculum. Findings elucidate the nuances associated with implementing an MBI in a rural middle school and have notable implications for development, scalability, and sustainability.

## Introduction

The onset of adolescence is paralleled by students’ progression to middle school and marks a particularly sensitive development period accompanied by entirely new social and cognitive demands. Students are expected to develop and sustain relationships with larger, diverse peer groups, maintain academic success, and take on more extracurricular responsibilities (Fallin et al., 2001; Hofferth et al., 2009; Ramírez, 2009). Simultaneously, hormonal fluctuations elicit changes in motivation, decision-making, and risk-taking behaviors (Eccles et al., 1993; Dahl, 2004). Students struggle with these challenging social interactions face a higher risk for friendlessness, rejection, and victimization (Kochenderfer-Ladd, 2004; Sandstrom, 2004; Zimmer-Gembeck et al., 2011).

Amidst this coupling of psychophysiological sensitivity to increasingly demanding sociocultural influences (Grosbras et al., 2007), middle-school students identify school as their primary stressor (American Psychological Association, 2013), and teachers report high levels of student anxiety (Flannery, 2018). Anxiety disorders are also most likely to emerge during the transition to middle school (Beesdo et al., 2009). This anxiety exacerbates adolescents’ efforts to manage interpersonal conflict (Marshall et al., 2015) and relationships (Epkins and Heckler, 2011), and lowers their academic performance (Mazzone et al., 2007). Thus, effective intervention is needed to mitigate the harmful impact of student anxiety on social, cognitive, and academic functioning. Additionally, although mental health disorders affect teens at equally alarming rates across urban and rural communities (Lambert et al., 2009; Merikangas et al., 2010), students in rural areas are less likely to access professional mental healthcare (Lambert et al., 2009). A shortage of services, health insurance, public transport, and internet, in addition to cultural and financial barriers, contribute to significant disparities in healthcare utilization among rural communities (Lambert et al., 2009; Douthit et al., 2015). School-wide mental health educational initiatives bypass barriers for obtaining services in traditional healthcare settings in rural communities, thereby offering a more equitable approach to mental health promotion.

Mindfulness-based interventions may offer a preventative solution to reducing students’ mental health pathology and improving academic functioning. Kabat-Zinn (1994, p. 4) defines mindfulness as “paying attention in a particular way: on purpose, in the present moment, and non-judgmentally.” Thus, MBI’s employ a variety of activities, typically over 8 weeks, to help foster this type of awareness, such as body scans, breathing exercises, meditation, and mindful movement (e.g., yoga) that help individuals connect compassionately with themselves (e.g., emotions, bodily sensations, thoughts) and their environment (Cullen, 2011). Exercises employed by MBIs generally teach: (1) attention regulation; (2) body awareness; (3) emotion regulation; and (4) change in self-appraisal (Hölzel et al., 2011). According to recent meta-analyses and reviews, school-based MBIs implemented among children are associated with improvements in psychological wellbeing (e.g., stress, emotion regulation), cognitive functioning (e.g., attention, working memory, and executive functioning), and academic performance, as well as reductions in self-harm behaviors, aggression, and intent to use drugs (Meiklejohn et al., 2012; Zenner et al., 2014; Maynard et al., 2017; Carsley et al., 2018; Suárez-García et al., 2020; Koncz et al., 2021; Liu et al., 2023). However, among existing school-based MBIs, many vary extensively in their curriculum, delivery, session frequency, and program length (Semple et al., 2017; Porter et al., 2022). Surprisingly, some of the most widely disseminated school-based MBIs have never been evaluated in rigorous randomized control trials (RCTs; Semple et al., 2017). Among the school-based MBIs that have been rigorously tested through RCTs, not all evaluations have been positive. One evaluation of a school-based MBI indicated that fourth and fifth grade girls in the intervention group reported higher perceived stress after the intervention compared to girls in the control group (White, 2012). Additionally, Britton et al. (2014) found no significant differences in middle school students’ internalizing and externalizing problems, attention problems, or self-reported mindfulness following a mindfulness meditation intervention compared to a control group. These inconsistent findings may be due to developmental influences that make middle-school aged children less likely to benefit from mindfulness. A meta-analysis conducted by Carsley et al. (2018) revealed that the effectiveness of MBI’s varied by age, where those in late adolescence (15–18) benefited more from the interventions compared to those in middle childhood (6–10). This is consistent with past theory that states that the effectiveness of mindfulness trainings may vary based on developmental periods, because plasticity of certain areas of the brain that can be altered by mindfulness changes across childhood (Roeser and Pinela, 2014). Porter et al. (2022) argue that MBI’s should take into consideration differences across developmental periods by tailoring the type of mindfulness practice, session length, and session frequency to best fit the specific needs of children at certain developmental periods. Thus, further research is essential to examine the acceptability, feasibility, and effectiveness of MBI programs delivered in middle schools, as adaptations to MBIs may be needed to fully address the psychological functioning of this particular age group.

Teacher-led MBIs may provide unique benefits for students. A meta-analysis found that teacher-led social and emotional learning programs were associated with better academic outcomes when compared to programs led by outside personnel (Durlak et al., 2011). Another meta-analysis of MBI’s also demonstrated that teacher-led MBI’s were more effective at improving cognitive and emotional outcomes compared to MBI’s led by outside personnel (Carsley et al., 2018). A successful implementation of a teacher-led MBI in high school was the program Learning to Breathe (Frank et al., 2021). This MBI implemented among high schoolers led to improvements in executive functioning, emotional awareness/clarity, stress, impulsivity, substance use, and self-compassion. Despite this success among high schoolers, there are mixed findings regarding the effectiveness of teacher-led MBI’s among middle schoolers (Carsley et al., 2018).

Domitrovich et al. (2016) suggest that although social and emotional learning programs are designed to target students, they may also have benefits for the teachers who are implementing such interventions. The authors did indeed find that the intervention being evaluated had a positive effect on teachers’ self-efficacy, burnout, and socio-emotional outcomes. Thus, theoretically, an additional advantage of teacher-led MBIs is that it gives teachers the opportunity to learn and integrate mindfulness into their own lives. Teachers describe their occupation as stressful and student misbehavior is a significant factor (Klusmann et al., 2008; Gray and Taie, 2015). MBIs may help improve teacher’s self-efficacy to handle job-related responsibilities and challenges, which, in turn, is associated with job satisfaction and student achievement and behavior (Caprara et al., 2006). Targeting teachers in the delivery of school-based MBIs may have direct benefits on teacher wellbeing, and these benefits may ripple down to their students.

Despite the unique benefits of having teacher-led MBIs, limited research has focused on gathering qualitative data regarding the acceptability and feasibility of such programs, particularly in rural communities. This is important given that teachers’ comfort with implementing programs has been associated with better program outcomes (Parker et al., 2014; Bazzano et al., 2018). Dariotis et al. (2017) conducted one of the few qualitative analyses to date of feedback gathered from students and teachers after the implementation of a school-based MBI. The authors found that teachers valued communication and collaboration with program implementers in order to successfully ensure program integration. However, no feedback has been gathered from teachers attempting to implement MBIs themselves. Feedback from teachers and students is necessary to create effective and sustainable interventions. This is especially true for rural areas given the unique barriers interventionists may face when working with these communities.

The present study evaluated a teacher-led MBI that was implemented among sixth-graders in a rural middle school using a qualitative methods approach. Delivery of the MBI was coordinated by mindfulness experts and the authors’ conducted an independent evaluation of the educational curriculum. The authors’ primary research aim was to assess the feasibility and acceptability of the curriculum based on teacher and student feedback. Written and verbal feedback was gathered from teachers and students during and following curriculum trainings.

## Materials and methods

### Participants and procedures

Using an exploratory qualitative methods approach, the present study evaluated the acceptability and feasibility of a teacher-led MBI delivered through 10 weekly sessions at a rural middle school in a rural town in the pacific northwest (town population of approximately 10,000). Approximately 500 total children were enrolled in this school, including children in grades 6th through 8th (i.e., approximately 11–14 years of age), and about 64% of these students qualified for free/reduced lunch. This particular school also includes one resident school counselor, who is available for student needs as they emerge. The principal from the school was interested in implementing the curriculum in his classrooms, in collaboration with the developers of the *AttuneEd*^®^ (curriculum, who were local psychologists or social workers and mindfulness experts. Prior to obtaining approval from an university Institutional Review Board (IRB), the research team, who were conducting an independent evaluation of the curriculum, received permission from the principal to collect data at each step of their pre-planned implementation delivery process, including eliciting feedback regarding the curriculum. Qualitative data regarding the acceptability and feasibility were collected from teachers and students in 2018. All procedures were approved by an university IRB.

Participants included sixth-grade students (*N* = 160), all middle school teachers, and the school counselor (*N* = 30). Boys comprised 41.9% (*Mage* = 11.5 ± 0.5 years) and girls comprised 48.8% of the sample (*Mage* = 11.4 ± 0.5 years). The student sample was predominately non-Hispanic white (56.3%), with Latinx/Hispanic (8.1%), Black/African American (0.6%), Native American/American Indian (0.6%), Asian (0.6%), Bi/Multiracial (6.9%), and Other (6.9%; e.g., text response indicating “American”) comprising the rest of the sample; 20.0% of the sample did not report their race/ethnicity. Demographic data from the teachers were not collected.

Three of the school’s physical education (P.E.) teachers enthusiastically agreed to co-deliver the mindfulness curriculum during their existing health education classes with the assistance of the curriculum developers. All other teachers and staff were encouraged, but not required, to reinforce the core principals of the intervention throughout their classes. Passive parental consent was obtained prior to data collection from students. A brief description of the study was sent home to the parents of sixth graders, and parents could decide to opt their child out of the study (i.e., not complete surveys). Six parents/caregivers opted their children out of the study. Students who did not have parental consent were provided with survey packets containing an identical cover page followed by blank pages with a note instructing them to spend the next few minutes relaxing or doodling. Financial compensation was not offered to students for their participation in the study. In consultation with the principal, it was agreed that an appropriate acknowledgement would be a post-intervention celebration for the students (including food, drinks and erasers as gifts) and an $800 gift to the P.E. department to purchase new equipment. The monetary gift was given regardless of the survey completion rate from teachers and students. Written consent was obtained from teachers who provided written feedback. The school requested that trainings with teachers be recorded so that they have these resources for future implementations of the intervention. A waiver of consent was obtained from the IRB because: (a) training sessions would be recorded by the school regardless of our research team’s involvement, (b) video recorders were faced toward the interventionists only so that footage could not be used to identify a single individual, (c) no data obtained would be linked to any identifiable information, and (d) analysis of the recorded data would not adversely affect the rights or welfare of the subjects, nor their relationship with the school. Prior to all trainings, teachers were informed that their verbally expressed opinions may be used to modify the mindfulness curriculum, and may be reported in aggregate, for research purposes. Students who provided quantitative and qualitative feedback were not asked for written assent.

### Procedures

#### Intensive trainings for P.E. teachers

Three P.E. teachers attended two full-day intensive trainings in which they reviewed the *AttuneEd*^®^ (curriculum. Both sessions were led by one of the curriculum developers. The first full-day training occurred before the intervention started and covered content for the first five sessions. The second full-day training occurred in the middle of the intervention and covered content for the second five sessions. These trainings were audio and video recorded. Verbal and written feedback was also solicited during each session. P.E. teachers were compensated $40 for each session (up to $80 total).

#### Brief trainings for all teachers

Within a week of each intensive training, two separate 1.5-h professional development workshops were conducted with all teachers. Core curriculum terms and exercises were reviewed and teachers. Written feedback was solicited from teachers after this meeting. Teachers who completed the brief trainings and agreed to participate in the current study received $10 for providing feedback after each training session (up to $20 total).

#### Student and teacher acceptability qualitative feedback

Students provided written responses to four open-ended questions at the end of the 10th lesson. P.E. teachers provided written responses to eight open-ended questions regarding the feasibility and acceptability of each lesson in the curriculum. All other teachers provided written responses to five open-ended questions at the end of each brief training workshop. Sample items include the following: “What did you like about the new classes?” (students); Are there any terms or words we used that bug you or you could imagine bothering other teachers or students? “If so, what words might you suggest we use instead?” (P.E. teachers) and; “What barriers could you imagine coming up when delivering these exercises?” (other-subject teachers). See Table 1 for all questions that teachers and students were asked. Items were created by the study team.

**TABLE 1:** Teacher and student feedback questions.

P.E. teacher questions
1. Are there any questions regarding the content we have reviewed thus far? What points could be made clearer?
2. Are there any terms or words we used that bug you or you could imagine bothering other teachers or students? If so, what words might you suggest we use instead?
3. What are your thoughts regarding the review of the anatomy and physiology behind this session?
4. Was the information regarding the research behind this lesson helpful? Would you suggest covering more or less research, or covering it in a different way?
5. Do you have any suggestions for improving the organization or structure of the information and exercises presented in this lesson?
6. How do you imagine it will go to teach this lesson to your students?
7. What barriers could you imagine coming up when delivering this session?
8. Do you have any additional suggestions for helping improve the delivery of these lessons in the classroom?
**Other-subject teacher questions**
1. Are there any questions regarding the content or exercises we have reviewed today? What points could be made clearer?
2. Are there any terms or words we used that bug you or you could imagine bothering other teachers or students? If so, what words might you suggest we use instead?
3. What barriers could you imagine coming up when delivering these exercises?
4. Do you feel comfortable leading a mindfulness activity or would you prefer it to be prerecorded?
5. Do you have any additional suggestions for helping improve the delivery of these exercises in the classroom?
**Student questions**
1. What did you like about the new classes?
2. What did you not like about the classes?
3. Did your teachers do any of the exercises in your other classes? If so, how many? Did you find them helpful? If so. How do, or what did they do for you?
4. The new classes will be delivered to incoming sixth grade students next year. Do you have any recommendations for making the classes or exercises better?

Physical education (P.E.) teachers responded to the 8 questions above following review of each individual lesson with the interventionists. That is, P.E. teachers responded to 40 questions at each training, resulting in 80 questions total. Other-subject teachers responded to the 5 questions above once at the end of each training workshop, resulting in 10 total questions. Students responded to 4 questions after the final lesson in the intervention.

#### Student quantitative acceptability ratings

After the last intervention session, students were asked to respond to five researcher-created acceptability items on a five-point Likert-type scale from 1 (*strongly disagree*) to 5 (*strongly agree*). Items included: “I gained something positive from these classes”; “These classes made me feel worse,” “Because of these classes, I understand myself better; my thoughts, feeling and body”; “Because of these classes, I understand other people better”; and “Overall, I am happy with these classes.” Items were created by the study team.

### Curriculum description

The MBI curriculum, called *AttuneEd*^®^ (Allen and Tang, 2019), included 10 weekly sessions and was designed to be flexible in terms of session duration and context of delivery, so that teachers can adapt the curriculum to the unique needs of their school. The curriculum was broken into two sections of five sessions each (Table 2). The first section, *self-regulation*, focused on key skills and strategies to regulate emotions, including increased awareness of thoughts, feelings and body, as well as a focus on breathing. The second part of the program, *interpersonal awareness*, built on this foundation and taught students how to use their self-regulation strategies to navigate interpersonal relationships. Each session included mindfulness-based activities and a brief lesson in anatomy and physiology, and could be delivered in as few as 20 min. Feedback gathered during the intensive trainings with P.E. teachers were used to adapt the curriculum before it was delivered to students. For example, certain images were removed/replaced due to teacher’s concerns that students or their parents would find them unacceptable. Additionally, the number of slides provided to teachers were reduced.

**TABLE 2:** *AttuneEd*^®^ program curriculum.

Lesson	Fundamental skills for self-awareness	
1	Relationship with observer-self	Learn to reliably access and strengthen the innate ability to calmly observe thoughts, emotions and physical sensations in order to gain cognitive flexibility in responding to them
2	Relationship with breath	Learn that breath is a powerful tool to help regulate our physiology and focus our mind
3	Relationship with emotions	Learn skills necessary to sit with difficult emotions and learn to respond rather than react
4	Relationship with thoughts	Learn how to observe our thoughts objectively, without being misled by them
5	Relationship with body	Increase awareness of times when we over or under identify with our bodies and explore ways to develop a compassionate relationship that includes listening to the body and treating it well
	**Mindfulness practices interpersonal awareness**	
6	Basics of interpersonal neurobiology	Learn how interconnected our nervous systems are; we can help calm or excite a social situation with the way we choose to move, speak, and breath
7	Empathy and compassion	Learn skills to understand the inner lives of others and how to take multiple perspectives
8	Mindful communication	Explore mindful communication in order to regulate ourselves before we communicate
9	Mindful listening	Learn to listen with non-judgmental curiosity and become aware of when our thoughts and emotions inhibit our ability to hear others’ perspective
10	Interconnection with the broader world	Explore how our actions affect each other and the broader world around us–what we eat and do affects us and the world around us

In the current study, lessons were delivered weekly on Fridays, during sixth-grade P.E. classes. There were six P.E. classes each day. During the first three classes of the day, P.E. teachers observed the curriculum developer(s) as they delivered the lessons in order to increase their familiarity and comfort with the content. During the last three classes of the day, P.E. teachers were expected to co-lead delivery of the lessons. A school counselor was available to students if they experienced any significant distress during delivery of the curriculum. The 10-lesson curriculum was delivered in Spring 2018 over a span of 12 weeks. Due to the school schedule, there was a 1-week break in-between lessons three and four and lessons five and six.

### Data analytic plan

Audio recordings from both intensive training sessions for P.E. teachers were transcribed verbatim. Written feedback from P.E. teachers, all other teachers, and students were copied and organized into excel files. Transcribed recordings and written feedback were double-coded by two members of the research team using a combination of inductive and deductive coding (Fereday and Muir-Cochrane, 2006). The coders and analyses were trained and mentored by a researcher with extensive experience in qualitative data collection and analyses. The analysts were master’s level members of the research team (AN and GW) who have conducted qualitative data analyses prior to this study. Researchers first used an inductive approach by reading all transcriptions and recording reoccurring themes. These themes were then condensed and organized using a deductive approach guided by identified themes. Two researchers coded each transcript, then met to discuss discrepancies and came to an agreement on coding. Both coders maintained an inter-rater reliability of at least 80% throughout the coding process. Coded qualitative data were then organized into meaningful themes and subthemes. Coded responses to each of the questions from the brief training workshops were then categorically re-coded to indicate whether teachers found the lessons to be: more or less feasible, and more or less acceptable. These codes were then used to calculate proportions of teachers that endorsed a particular aspect of the intervention (e.g., acceptability of language). For the student acceptability items, descriptive statistics were calculated.

## Results

### Acceptability and feasibility qualitative findings

Data analysis of students’ and teachers’ free-responses, and transcriptions of teachers’ audio recorded feedback revealed three major themes from teachers: (1) Cultural Considerations, including the need for this type of program, as well as the need for adaptations to increase student and community acceptability; (2) Teacher Self-Efficacy, including teachers’ desire for more support in content delivery; and (3) Barriers and Facilitators to Student Acceptability, including students’ perspectives on content delivery and intervention success. In the following sections, we describe each theme, referencing both student and teacher perspectives.

#### Theme one: cultural considerations

The first theme that emerged was related to middle-school culture, including the need for this type of program in their school. Teachers expressed a strong desire for support in managing student stress and peer dynamics:

*I just want to comment to this too because we have like this—these girl group, not gangs I would say, but they’re really mean, really mean girls and it’s like they cannot figure out for the life of them how to get along with each other and how to not be all up in each other’s business*…*it’s just so timely, ha. It’s always timely with girl drama but you, you know, it just really seems like it’s flaring up*… (verbal feedback, P.E. training 2).

Teachers also noted the need for adaptations to increase student and community acceptability. Mainly, several teachers expressed some concern with language prior to implementation of the first part of the curriculum:

*“I did not have any words that bothered me, but I do run into the idea of ‘mindfulness’ does not go over well with some of my students”* (written feedback, P.E. training 1).

Other teachers noted areas that may be met with resistance:

*“The observer-self wheel might meet some resistance to a conservative religious listener. Explaining the imagery and how we will use it will be important”* (written feedback, P.E. training 1)

*“keep it scientific vs. spiritual” (written feedback, PE training 1*).

*“Kids are pretty concerned about body image and the changes they experience at puberty. Very stressful for them”* (written feedback, P.E. training 2).

Another teacher commented on the diversity of student backgrounds within their school, and expressed concern that the intervention could employ more sensitive language around family structure.

*“Careful w/cultural barriers in classrooms! Lots of kiddos don’t know families/’self-identity’ and feel upset showing it (Foster kiddos etc.)”* (written feedback, workshop 2).

Teachers also expressed concerns about student perceptions of the terminology and concept of “window of tolerance”:

*“I don’t struggle, but I could see students mocking some of the terms, such as ‘windows of tolerance.’ If we use it enough though, it will normalize”* (written feedback, workshop 1).

On the other hand, teachers unanimously found the anatomy and physiology portions of the lessons to be meaningful and critical to creating student buy-in to the curriculum:

*“I believe [anatomy and physiology] is a critical component to helping create buy-in to curriculum and how it will benefit the student”* (written feedback, P.E. training 1).

#### Theme two: teacher self-efficacy

The second theme that emerged was related to teachers’ concerns with implementing the curriculum themselves and centered around teachers’ ability to manage student reactions in the classroom.

*“I think this will be one of the more difficult lessons to teach to get kids to grapple with thoughts”* (written feedback, P.E. training 1).

*“Well just the way you described anchoring is really helpful because it’s really easy and I mean it’s really familiar to you but the way you described it like I’m thinking how am I gonna remember her words to know to say that to kids”* (verbal feedback, P.E. training 1).

Concern about students’ ability to be vulnerable and teachers’ ability to manage related classroom dynamics and students’ emotions stayed constant from training 1 to 2. As one teacher noted,

*“Mindfulness requires a level of vulnerability in order to be successful. I fear that with a portion of students being vulnerable, a different portion would seize the opportunity to exert power (negative power) over their classmates”* (written feedback, workshop 1).

In dealing with student mental health, teachers wanted to ensure that roles and boundaries were not blurred:

*“One reason why a lot of these things are really difficult, is that line between going in depth and also not being like uh a therapist”* (verbal feedback, P.E. training 2).

*“Not necessarily a barrier but it could bring stuff up for students. Would be good to know they have a safe place to go to”* (written feedback, workshop 2).

Other teachers noted the importance of providing resources for students with learning difficulties:

“*With our # of kids on IEP* + *ELL I hope all terms have synonyms* + *visuals*” (written feedback, workshop 2).

Teachers desired on-going support for delivering the curriculum on their own, noting that delivery did not always get easier with practice:

*“I thought I did [feel comfortable leading activities], but only led them a few times and felt less comfortable as we went, so I guess I’d prefer it prerecorded*” (written feedback, workshop 2).

Many teachers requested specific resources to assist with curriculum implementation, including guidelines for adapting each lesson to their individual class groups; audio and video supplements; reference cards for mindfulness exercises; and visual aides to put up around the school.

*“Video of a leader/group of students doing an exercise”* (written feedback, workshop 1).

*“I like the option of watching/learning & teaching w/help, then once I become comfortable branching out on my own”* (written feedback, workshop 2) *“Maybe give us a cheat sheet of if we notice this - then try that. I just feel like I need more training*… *But I love what you’ve done so far!!”* (written feedback, workshop 2).

#### Theme three: barriers and facilitators to student acceptability

Overall, students perceived the mindfulness lessons to be acceptable:

*“It was a fun way to express yourself,”*

*“They were fun and got me ‘reunited’ with my friends and when it started we were fighting,”* and

*“It’s awesome how we get to share our feeling with one and another.”* Alternatively, when students were asked what they disliked about the curriculum, they responded with:

*“I didn’t like all the talking I just wanted to start on the activities,”*

*“Some of the work is hard,”*

*“Yoga,”*

*“That we mostly just sat there the whole time, and that it’s over,”* and *“Made me stressed and mad.”*

Students also reported a preference for their own community members to lead the mindfulness lessons. Some commented that they disliked the “*new people*” (written feedback, 6th grader) and that the interventionists were “*artificially upbeat and happy*” (written feedback, 6th grader). P.E. teachers also commented on this aspect of the lessons:

*“if a kid has a relationship with a teacher and it is infused in the class, and it’s brought up that way, it’s gonna have a different feel to it”* (verbal feedback, PE training 2).

*“I think coming in as somebody who they don’t have a relationship with and talking about this versus it being like sort of everybody talking about it together who are part of the school community is totally different*” (verbal feedback, interventionist, P.E. training 2).

Teachers also commented on student acceptability. For instance, some teachers noticed that their students enjoyed and benefitted from the relaxation exercises:

*“I thought the progressive relaxation—the kids always love that I mean because that is like the one time in the day that they actually do stop and they—it gets really quiet, even the chatties and even, you know, a couple kids are so relaxed, they fall asleep. And I still think that’s ok*” (verbal feedback, P.E. training 2).

Teachers also commented on the unexpected acceptability and frequent use of the “window of tolerance” concept in their classrooms:

*“I thought I didn’t like the ‘Window of tolerance’ the first time you were here. It’s grown on me and I like it now:”* (written feedback, workshop 2).

Alternatively, many teachers expressed concern about students’ discomfort with body image when learning about the yoga components:

“*Kids might be self-conscious of being in front of the class depending on how the room is arranged”* (written feedback, P.E. training 1).

“*Body image subject matter can be very sensitive, necessary but sensitive. Yoga changing physiology – that topic might meet resistance*…*most likely very few though. Need to keep if science less spiritual”* (written feedback, P.E. training 1).

“*Probably ned to set up ground rules. Might feel silly. Worry that they look silly. Comments by others. Poses might make them feel awkward at first”* (written feedback, P.E. training 1).

### Student and teacher acceptability quantitative findings

Acceptability ratings (range 1–5) suggested that students were happy with the classes (*M* = 3.72, *SD* = 0.96); that they gained something from the classes (*M* = 3.71, *SD* = 0.98); and that the classes helped them understand themselves (*M* = 3.26, *SD* = 1.24) and others better (*M* = 3.44, *SD* = 1.05). Student agreement was low for “classes made me feel worse” (*M* = 2.08, *SD* = 1.13); of note, 13 students responded “strongly agree” or “agree” to this question.

Most (63%) teachers expressed comfort leading a mindfulness activity independently following the first brief training; this percentage decreased following the second brief training to 31.6%. Relatedly, only 11.1% of teachers stated that they were not comfortable leading a mindfulness activity after receiving the first brief training. After the second training, 36.8% of teachers wanted more training before implementing intervention content independently and 15.8% wanted prerecorded exercises. Following the first and second brief training, 40.7 and 26.3% of teachers were worried about student participation (not wanting to look silly, not taking it seriously, students sabotaging the lessons for others) as a potential barrier, respectively, and 21.1% thought that the curriculum activities may increase distress among students.

## Discussion

Despite the wealth of literature outlining the positive impacts of MBIs, most of these studies have focused on adults (Teasdale et al., 2002; Baer, 2003; Shapiro et al., 2006; Hölzel et al., 2011; Hoge et al., 2013). Published interventions targeting children provide very little discussion of age-appropriate modifications (Burke, 2010; Zenner et al., 2014; Gould et al., 2016). Meanwhile, studies examining the acceptability and feasibility of MBIs implemented among rural schools are non-existent, creating a lack of knowledge on how to better design these programs to fit and address the cultural needs of these schools. Both of these considerations need to be addressed in order to improve the acceptability, feasibility, effectiveness, and, ultimately, sustainability, of MBIs implemented with these specific populations. When developing the current curriculum, the creators anticipated, for example, a need to integrate mindfulness-based perspectives into interpersonal contexts, given the importance of peer relationships to adolescents (Fuligni and Eccles, 1993). The entire second half of the described curriculum focuses on empathic, mindful and respectful communication. Feedback from teachers in the current study supports the need for this emphasis. Future work is need to evaluate whether school-based MBIs are effective in improving peer dynamics and whether these changes mediate additional improvements in students’ behavior and mood.

### Student’s and teacher’s feedback regarding acceptability and feasibility

Teachers in the current study suggested that teacher, student, and parent acceptability would be improved if terms and images which might conjure up thoughts of spirituality or religion were replaced with something more widely palatable such as a focus on the scientific aspects of mindfulness. Developing a manual with more universal language may be particularly important for communities in which mental health stigma is more pronounced, such as those living in rural areas (Hammer et al., 2013; Stewart et al., 2015). Additionally, it was suggested that language in the curriculum be more attentive to diverse gender identity and family structures. Teachers also reinforced the value of discussing the anatomy and physiology of mindfulness, noting it will improve student buy-in and map onto various student-learning outcomes. For example, including corresponding lessons in anatomy and physiology may allow teachers to replace several hours of current school curriculum, thereby enhancing the flexibility of program delivery while minimizing teacher burden. Student engagement in school-based MBIs is very low when presented as an in- or after-school elective (Foret et al., 2012). Considering the high rates of teacher burnout (Ingersoll et al., 2014), it is vital that perceived burden regarding the implementation of school-based MBIs remain low. Integrating MBIs into schools’ existing structure and curricula seems like the optimal approach to minimize burden and maximize reach.

One of the biggest challenges of bringing mindfulness-based mental health curricula into classrooms was teachers’ concerns related to handling student emotions. In particular, teachers expressed concerns that the curriculum exercises would bring up difficult emotions and traumatic memories for their students. In response to these concerns, interventionists: (1) modeled how to set the stage for appropriate sharing; (2) demonstrated how to express and respond to emotions in a validating manner; and (3) distinguished teachers from mental health professionals. This began with talking about rules related to confidentiality, which were similar to those used for sexuality education discussions (e.g., do not overshare; limits of confidentiality for minors). Students were also given a document outlining resources available if additional emotional assistance was needed; mental health treatment was normalized and likened to medical care from a physician. Teachers were encouraged to share their own emotional experiences and to model doing so in terms consistent with the curriculum (e.g., “My window of tolerance is low today, so I am going to take a few breaths at the beginning of class to help stay calm”). Finally, interventionists clarified the role of the curriculum—to teach basic mental health education and skills—and devised a plan (e.g., use grounding activities, doodle during exercises, stretch in the back of the classroom) when students reported feeling overwhelmed or appeared to become overwhelmed. During the delivery of this curriculum, it became evident that emotional expression and validation represent fairly significant shifts in conceptualizing students’ experiences and behaviors. It is important for future school-based MBIs implemented among middle school students to address these concerns, especially given that a significant proportion of this population has experienced a traumatic event (Gilbert et al., 2015). In order for teachers to feel confident in delivering trauma-sensitive skills in the classroom, dedicated training and ongoing teacher support are needed. In addition, schools should have systems in place to support the mental health of students who have significant emotional regulation issues (e.g., as one teacher noted, designating a “safe space” or person for children to utilize in order to help manage/process adverse feelings that may arise).

In addition to not being mental health professionals, most teachers are not mindfulness experts. This is reflected in feedback as teachers stated that various audio, visual, and physical supplements (e.g., pre-recorded lessons, posters, mindfulness reference cards) would help with implementation. Having more materials for teachers to reference may improve their comfort with program material and implementation. Relatedly, delivering school-based MBIs via digital platforms may help reduce teacher’s concerns delivering program material. Digital platforms might make the curriculum more accessible to teachers, while also improving the ability to disseminate the curriculum to a wider audience. This approach may be particularly useful in reducing geographic-specific inequities in access to mental health resources. It may also provide an opportunity to provide teachers with additional support through the use of online professional learning communities, which have been shown to engage teachers, increase their knowledge, and modify their teaching practices (Blitz, 2013). Future research may consider evaluating teacher’s effectiveness in delivering MBI’s prior to implementation. For example, Griffith et al. (2021) designed an assessment tool to assess the competence of individuals who lead such interventions, which helps to also provide helpful feedback to teachers and enhance the quality of the delivery of MBIs.

Based on the qualitative findings, students seemed to find the program to be fun and relaxing, demonstrating generally high acceptability. Relatedly, student engagement was high throughout the delivery period. Only one student did not participate in most activities and reported that the content was somewhat distressing. When given the option of going to another classroom, this student chose to stay in class and listen, but not participate in the exercises. Although the overwhelming majority of students enjoyed and found the curriculum helpful, several expressed discomfort with outside personnel delivering the sessions and teachers were aware of these sentiments. According to a meta-analysis of universal school-based socioemotional learning programs, teacher-led programs were associated with better academic outcomes when compared to programs led by outside personnel (Durlak et al., 2011). These data underscore the need to identify school-based MBIs which can be easily facilitated by teachers. In taking this approach, greater attention to the direct training and continued support of teachers is vital to facilitating self-efficacy for program delivery. Indeed, while it was the original plan to have P.E. teachers first observe and then lead curriculum lessons, curriculum developer(s) ended up serving as co-leaders after these teachers requested their continued support.

Notably, approximately 8% of students reported that they *agreed* or *somewhat agreed* that participating in the intervention made them feel worse. One potential explanation for these students’ experiences is the increase in self-awareness that mindfulness-based practices intentionally develop. Schonert-Reichl and Lawlor (2010) hypothesized that such self-awareness potentially leads adolescents to engage in critical evaluations of themselves and their emotional experiences. It has been suggested that the benefits of mindfulness-based exercises, including reductions in critical self-awareness and increases in wellbeing, are acquired with lengthier practice (Greenberg and Harris, 2012). The duration of the current program was short and additional studies are needed to identify the optimal intervention length for school-based MBIs. Notably, data collection for the student acceptability items occurred near the end of semester, when final exams were being administered. It is possible that the time frame of this assessment might have confounded students’ perceptions of the program’s effects on their mood. Nonetheless, future studies should continue to evaluate the potential for mental health education programs to inadvertently promote reductions in student wellbeing. It would be valuable to clarify whether certain components of school-based MBIs are problematic and to identify teachers and students who might be at risk for iatrogenic outcomes. All of this information would inform programmatic and school system-level modifications to minimize student and teacher harm and maximize their wellbeing.

### Future directions

The current study presents useful data regarding the feasibility and acceptability of a teacher-led mindfulness-based mental health education curriculum designed for middle school students in a rural town in the pacific northwest. Acceptability data for such interventions are rarely reported yet are necessary for successful implementation and sustainability. Importantly, because the study team was invited to collect data shortly after the school opted to deliver the described MBI, there was not adequate time to ground the current study’s assessment procedures in formal theories or frameworks related to implementation science. For example, some frameworks put forth helpful methods for assessing fidelity to identify the degree to which the intervention was implemented as intended (Ridde et al., 2020). Future research should integrate implementation frameworks and theories in order to inform a more comprehensive evaluation of the feasibility, acceptability and effectiveness of MBIs delivered in schools. Additionally, this study is affected by both mono-method and social desirability biases, meaning only self-report data were collected. Future studies should consider integrating multiple data collection methods, such as classroom observations, to help mitigate these biases.

One of the most significant limitations of the current study’s approach relates to the limited generalizability of the study’s findings. The opinions of one rural school’s students and teachers are presented. Although few demographic details were collected from participants, they all resided in a rural town in the pacific northwest, a predominantly non-Hispanic white state. As such, it is unclear to what extent the views presented in this paper would be shared by others, including students and teachers with more diverse identities and from varying geographic locations. It is important that future research attend to such demographic variables given that mental health curricula may be need to be tailored to fit the needs of vulnerable and marginalized youth, such as transgender and gender non-conforming individuals, as well as communities with varying school resources.

Although the current study’s findings are limited in their generalizability, several recommendations are provided for consideration in order to help maximize the feasibility and acceptability of teacher-led MBI interventions. Future interventions should focus on designing programs that include more universal language, age-specific modifications, additional support for teachers and students, and strategies to further integrate program content into school culture. Considering the unique needs and cultures of each school system and student body, it may be beneficial to have some formal assessment of such programs at each individual school. More rigorous experimental designs, such as randomized controlled trials, are also needed to demonstrate the effectiveness of such interventions and mindfulness in general. Overall, mental health education in public schools should utilize a non-pathologizing, universal curriculum teaching basic concepts and best practices for emotional wellness. Additionally, targeted interventions for students who may need some scaffolding should be considered as should individual counseling for students who have higher level complex issues. Legislation around similar types of mental health education should consider these points as states begin to require the integration of mental health education into the existing school curriculum.

## Data availability statement

The raw data supporting the conclusions of this article will be made available by the authors, without undue reservation.

## Ethics statement

The studies involving humans were approved by the University of Oregon Institutional Review Board. The studies were conducted in accordance with the local legislation and institutional requirements. Written informed consent for participation in this study was provided by the participants’ legal guardians/next of kin.

## Author contributions

AF: Writing – original draft, Writing – review & editing. AN: Formal analysis, Writing – original draft, Writing – review & editing, Visualization. GW: Data curation, Formal analysis, Investigation, Writing – original draft, Writing – review & editing, Visualization. CG: Data curation, Investigation, Writing – review & editing. NK: Conceptualization, Funding acquisition, Investigation, Methodology, Project administration, Supervision, Writing – original draft, Writing – review & editing.
